# The Vitreous Ecosystem in Diabetic Retinopathy: Insight into the Patho-Mechanisms of Disease

**DOI:** 10.3390/ijms22137142

**Published:** 2021-07-01

**Authors:** Siva S.R. Iyer, Mollie K. Lagrew, Stephanie M. Tillit, Ramak Roohipourmoallai, Samuel Korntner

**Affiliations:** Department of Ophthalmology, University of Florida College of Medicine, 1600 SW Archer Road, Gainesville, FL 32610, USA; mollie.mansfield@ufl.edu (M.K.L.); stephaniemtillit@gmail.com (S.M.T.); ramakroohipour@yahoo.com (R.R.); korntner@ufl.edu (S.K.)

**Keywords:** vitreous, diabetes, retinopathy, cytokine, interleukin, eicosanoid

## Abstract

Diabetic retinopathy is one of the leading causes of blindness in the world with the incidence of disease ever-increasing worldwide. The vitreous humor represents an extensive and complex interactive arena for cytokines in the diabetic eye. In recent decades, there has been significant progress in understanding this environment and its implications in disease pathophysiology. In this review, we investigate the vitreous ecosystem in diabetic retinopathy at the molecular level. Areas of concentration include: the current level of knowledge of growth factors, cytokine and chemokine mediators, and lipid-derived metabolites in the vitreous. We discuss the molecular patho-mechanisms of diabetic retinopathy based upon current vitreous research.

## 1. Introduction

Diabetic retinopathy (DR) is a leading cause of blindness in the developed world [1]. The worldwide DR prevalence among individuals with diabetes is as high as 34.6%. It is estimated that DR affected approximately 93 million individuals globally in 2010. Furthermore, because the prevalence of diabetes mellitus is growing, particularly in low- and middle-income countries, it is expected that the incidence of DR will increase in the future [1]. Among individuals with diabetes, the annual incidence of DR ranges from about 2.2% to 12.7% depending on geographic region [2]. The prevalence of DR and its subsequent sequela is substantially higher in individuals with type 1 diabetes, increased diabetes duration, poor glucose control, and other comorbidities such as high blood pressure or high cholesterol [1]. Proliferative diabetic retinopathy (PDR) with complications such as vitreous hemorrhage and tractional retinal detachment (TRD) represents the most advanced stage of DR [3].

The vitreous is a near acellular and avascular body composed of 97–99% water [4,5]. Over 70% of the collagen in the vitreous is type II [5]. Hyaluronan (HA) is the major glycosaminoglycan (GAG) in the human vitreous [5]. As one might expect, the molecular make-up of the animal vitreous is not equivalent to the human vitreous. The components of the vitreous humor are not uniform or pattern-driven, even among animal species, and there are differences in concentrations of electrolytes, and GAGs [6]. For example, chondroitin sulfate is the predominant GAG in chicks rather than hyalauronin [7]. The vitreous cortex is identifiable in youthful vitreous gel, and with age, liquefaction of the vitreous body occurs [8,9]. Liquefaction occurs earlier in the diabetic eye [8]. However, the delay of complete posterior vitreous detachment sets the stage for structural damage to the retina as proliferative retinal disease progresses.

The vitreous molecular ecosystem in the diabetic eye is generally reflective of retinal disease and is an emerging science. Most of the literature currently available on molecular vitreous mediators in the human vitreous has been from the study of eyes with PDR. This is understandable as non-proliferative diabetic eyes undergo surgical intervention less often. Molecular study of the vitreous may seem a recondite science, but the importance of vitreous molecular constituents cannot be overstated because it helps us understand disease pathogenesis and processes. The vitreoretinal interface and micro-environment represent an intimate and complex relationship. In this review, we explore the current knowledge on vitreous mediators in DR and discuss their characteristics and known dynamics.

## 2. Challenges to the Study of the Vitreous

### 2.1. Approaches

The study of the vitreous structure is difficult because its ultrastructure can be altered with processing, leading to the introduction of artifacts. For example, the early assertion of chondroitin sulfate’s role in glycosaminoglycan (GAG)–GAG interactions with collagen crosslinking may have been due, in part, to artifactual findings [4,10]. Many types of techniques have been used to study the vitreous including whole dissections [9,11], electron microscopy with rotary shadowing [7], dark-field illumination [9,12], spectroscopy [13], and digestion by proteinases, to name a few [4,6,7,9,10,14]. Early gross human anatomical studies were that of cadaveric eyes [9], but the study of the molecular science of the vitreous cannot be conducted in this manner if the study is to reflect active and ongoing disease.

Most molecular studies are conducted after a vitreous sample is obtained [13]. Untargeted, proteomic-based approaches such as liquid chromatography tandem mass spectroscopy (LC-MS/MS) can provide the sensitivity and specificity needed to objectively analyze the vitreous [15]. For targeted analysis, enzyme-linked immunosorbent assay (ELISA) by far has been the most common way to detect and analyze vitreous cytokines, having replaced radioimmunoassay [16], but in the early 2000s, multiplex methods arrived which were more cost-effective as well as efficient in measuring multiple mediators simultaneously [17,18,19]. Methods using multiplex technology that enhance reproducibility and detect proteins in lower concentrations such as electrochemiluminescent (ECL) sandwich immunoassay and proximity extension assay (PEA) are now widely available [20]. These techniques allow many proteins to be analyzed simultaneously with as small as 1 µL of volume [20].

As human samples are taken from a small volume and generally require aseptic ocular surface conditions, they are most safely obtained in controlled clinical and surgical settings. Non-invasive techniques to study the vitreous from patients such as from vitreous reflux after intravitreal treatment have been recently described [17]. The majority of all vitreous samplings for molecular studies, however, are conducted in the surgical suite. The vitreous has to be removed without damage to the retina or other eye structures [21] and has to be cooled and stored immediately (usually on dry ice), transported appropriately, and then stored again (usually in the −30 to −80 C range) until analysis can be conducted [22]. Many vitreoretinal operations at academic centers are performed after hours and are often unscheduled. Therefore, it follows that from a technical and logistical standpoint, vitreous sampling has to be planned and performed meticulously. Infusion fluid dynamics prior to sampling and pre-treatment with a variety of intravitreal therapies can alter the natural state of the vitreous [3]. In addition, the sample obtained from one area in the vitreous may not represent the molecular environment in another area [23]. Herein, we describe our sampling technique for pure undiluted human vitreous in the surgical suite.

### 2.2. Human Vitreous Sampling Technique

Pure vitreous removal for the purposes of study should be conducted after institutional review board approval has been established and informed consent has been obtained. The vitreous can safely be removed under direct visibility with immediate access to intraocular pressure adjustment to avoid lens damage (posterior collapse), hemorrhage, and suprachoroidal fluid accumulation as the eye is decompressed. The vitreous cutter (27 gauge, 25 gauge, or 23 gauge) after priming (to remove fluid from the tubing) is a functional tool using aspiration and, in some instances, high cut rates (≥7500 cuts per minute) to obtain the sample with connection to a 1 mL tuberculin syringe handled by an assistant. The cutter can be maneuvered (with a careful cutter aperture orientation) to obtain a mid-vitreous sample or moved to different areas with care. In the case of a TRD, awareness of the location of the posterior cortex (sometimes very anterior in the mid-periphery) is important as cortical incision may result in unintended retro-cortical sampling. Using the technique above, approximately 1 mL of sample in most cases can be obtained from the human eye safely prior to significant decompression. Simultaneous air exchange to maintain eye volume while the sample is being removed may be more favorable in high-risk cases and in cases of pre-incisional hypotony.

### 2.3. Interpretation of Vitreous Constituents

**Figure 1:**
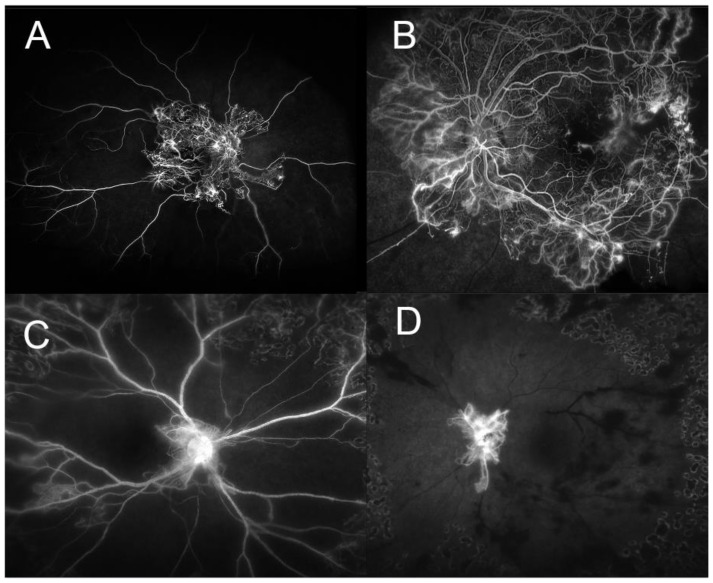
Angiographic evidence of the loss of normal retinal vasculature contributing to the breakdown of the blood–retinal barrier in uncontrolled diabetes mellitus. (**A**) Right eye of a 29-year-old African American female with peak arterio-venous phase showing neovascularization and no capillary perfusion beyond the posterior pole. (**B**) Same patient’s left eye showing an enlarged foveal avascular zone, and heavy neovascularization at the border of retinal perfusion and non-perfusion. (**C**,**D**) are from a 28-year-old African American female showing global retinal ischemia with little perfusion beyond the peripapillary vasculature. (Images courtesy of Siva S.R. Iyer, MD).

Although cytokine/chemokine levels are generally reflective of active processes, the correct translation of the diabetic vitreous language still eludes us. It is not completely clear if vitreous cytokines suggest localized processes or are reflective of a systemic process with soluble migration of cytokines to the vitreous. Active PDR can result in a loss of vast amounts of normal healthy retinal vasculature (Figure 1), begging the question if a substantial breakdown of the blood–retinal barrier hastens a two-way mechanism between the vitreous and systemic circulation.

Take, for instance, a common reason for diabetic vitrectomy in PDR: persistent vitreous hemorrhage. Although some studies distinguish the quality of the vitreous explicitly as containing a hemorrhage or not and alter inclusion criteria in the study design [24,25,26,27,28], not all do. Interestingly, even when vitreous hemorrhage has been excluded from study eyes, vitreal hemoglobin can still be detected in PDR and in control eyes [29]. Findings such as this introduce broader questions regarding the protein content in the vitreous. Protein normalization is often not known or performed for all studies, and this may affect the interpretation of what a given protein level truly reflects in the vitreous [26,29]. Additionally, scatter laser treatment can also have significant effects on cytokine levels in the vitreous [27,30,31,32]. Not all studies notate the amount or time interval of laser application prior to vitreous sampling [33].

A broad inclusion of diagnoses as controls likely does have some influence on results and can introduce confusion in the future, as more information is discovered regarding vitreous molecular markers in retinal disease. Although most controls always exclude diabetic eyes, some include epiretinal membranes [34,35] and/or rhegmatogenous detachments [36,37,38,39,40], while others only include the diagnosis of macular hole [26,41,42,43].

The location of vitreous sampling is not always clearly stated in the literature. It is often unknown if certain samples were obtained from different regions within the vitreous body or isolated to the mid-vitreous. Even mid-vitreous sampling notations rarely have anterior or posterior specific distance descriptions in relation to the lens or pre-macular area. The specific location is important, illustrated by varying glucose and VEGF concentrations in the vitreous, with higher concentrations found in the pre-macular vitreous compared to the mid-vitreous [23,44]. Bainbridge and colleagues have shown that oxygen tensions closer to the posterior pole are higher compared to the mid-vitreous or periphery in PDR [45]. Pre-macular oxygen tension levels correlated positively with mid-vitreous VEGF levels, but this correlation was not sustained when mid-vitreous oxygenation and VEGF levels were compared [45]. These concepts of qualitative details in vitreous sampling likely have some influence on the quantifiable data and make data interpretation more vulnerable to erroneous conclusions in disease patho-mechanisms.

## 3. The Diabetic Vitreous Environment

### 3.1. The Glycemic State

The systemic glycemic state can be characterized with the measure of glucose in the vitreous [46,47]. Post-mortem, glucose or glucose + lactate levels in the vitreous can indicate hyper- or hypoglycemic fatal states [46,47]. A vitreous glucose level of ≥234 mg/dL or a combined level of glucose + lactate of ≥427 mg/dL is the upper threshold in the determination of hyperglycemia in the body [46]. Intravascular exposure to high glucose, glycated and glycosylated products, and environmental stress such as hypertension can lead to pericyte loss and oxidative stress in diabetic retinopathy [48]. Glucose levels in the posterior region of the vitreous humor have been shown to be higher than the central or anterior regions of the vitreous humor in diabetics [44], and may be reflective of the metabolic process at the retina level. However, even prior to the vascular findings of microaneurysms and other microvascular changes found in clinical DR, pre-clinical neuro-degenerative findings can be found in DR and are thought to be induced through innate immunogenic and pro-inflammatory responses [49]. The data on the vitreous in non-proliferative diabetic retinopathy (NPDR) cases are very limited because of the difficulty of sampling in those patients who do not need vitrectomy. Those few studies that evaluated the level of VEGF in non-proliferative diabetic retinopathy are not conclusive [50,51,52]. No studies to date have found any conclusive evidence of reproducible vitreous mediators in pre-clinical DR human eyes.

### 3.2. Proteins in Diabetic Vitreous

There are many studies of the diabetic vitreous using a proteomic approach (sodium dodecyl sulphate–polyacrylamide gel electrophoresis (SDS-PAGE), mass spectrometry, liquid chromatography, etc.) [53,54,55,56]. This review aims to describe parts of the vitreous environment in diabetic retinopathy rather than list proteins found in the vitreous. However, it is important to mention the proteins in the diabetic vitreous (other than albumin) that have been recurrently identified across multiple studies using a proteomic approach. These include apolipoproteins (AI, AII, AIV) [53,55,57,58,59], α1-acid glycoprotein [53,55,57], clusterin [55,60], complement C3 [53,55,57,58], fibrinogen [55,58], α1-antitrypsin [55,58], prothrombin [53,55,57], and platelet derived factor (PEDF) [53,55,57]. The presence (or absence) of a protein does not necessarily imply a role (or lack of a role) in the pathogenesis of a disease. As it can be seen above, some of the most recurrently identified proteins are members of very different systems, implying that DR is not a single-path disease process, but one of cross-talk across a myriad of signaling pathways.

### 3.3. Animal Models

Although this review focuses on the human vitreous in DR, animal models have provided and continue to provide invaluable information about mediators in microvascular disease. The complexities of human vitreous sampling are at least partially circumvented in animal models without the necessity of a surgical suite and burdensome transport. Animal models have also afforded us the benefit of controlled experimentation in angiogenic states.

The discovery of VEGF as a distinct growth factor was obtained after conditioned media from primary bovine pituitary follicular cells were analyzed and shown to induce proliferation of vascular endothelial cells [61]. The binding location for VEGF was demonstrated to be endothelial cells in a rat model in the early 1990s [62]. Diabetes’ and VEGF’s deleterious effects on the blood retinal breakdown have been shown in a rat model to act via influences on occludin, an important tight junction protein [63].

Interleukin-8 (IL-8) has been shown to cause tubular morphogenic changes to bovine endothelial cells [64]. This change was stopped with inhibition, suggesting a pro-angiogenic role in the retina [64]. Tumor necrosis factor alpha (TNF-α) and interferon gamma (IFN-γ) show higher concentrations in vitreous PDR in rats compared to controls but show lower concentrations in the corresponding serum [65]. It has even been suggested through an animal model that the “normal vitreous” may be pro-angiogenic (with cell migration and proliferation enhancement) through phosphoinositide 3-kinase/protein kinase B (P13K/Akt) signaling via the receptor tyrosine kinase anexelekto (Ak1) activation, as shown using the bovine vitreous and human retinal microvascular endothelial cells [66]. These findings in animal biology help to reshape the ideas of what cytokines and their associated cascades might mean in the human eye.

### 3.4. Growth Factors and Their Roles

Growth factors respond to their changing environment via receptor signaling cascades that stimulate growth, proliferation, and/or differentiation [16,52,67]. Persistent hyperglycemia, as well as dyslipidemia, is a major risk factor leading to ischemic changes in the retina, ultimately stimulating the release of angiogenic growth factors promoting neovascularization [68]. Several growth factors, such as platelet derived growth factor (PDGF), transforming growth factor β (TGFβ), basic fibroblast growth factor (bFGF or FGF-2), and pigment epithelial derived factor (PEDF) have been described in the diabetic vitreous literature [42,67,69,70,71,72].

The most well-studied growth factor in PDR is the VEGF family, including placenta growth factor (PIGF) [73]. The source of PIGF is the retinal pigment epithelium [74]. The assertion that VEGF plays a role in retinal neovascularization was introduced after VEGF was shown, for the first time, to bind bovine retinal endothelial cells in an autocrine role in 1994 by Plouet and colleagues [75]. Since that time, considerable effort has been invested in the study of VEGF in retinal angiogenesis. VEGF is upregulated in many cell types in response to hypoxic conditions (with hypoxia inducible factor 1 (HIF-1) induction) including endothelial cells and the retinal pigment epithelium [22].

**Figure 2:**
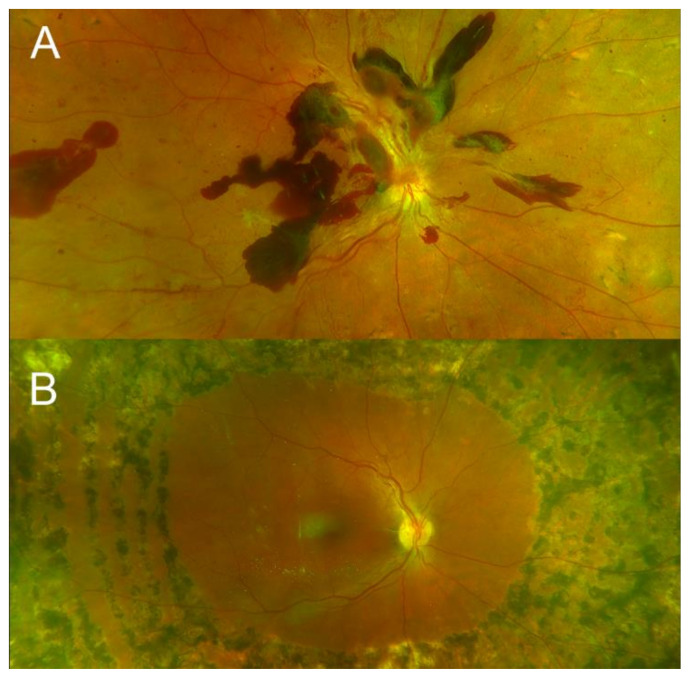
22-year-old Caucasian female type 1 diabetic (diagnosed age 11). Top image (**A**) shows scattered retro-cortical hemorrhage in the posterior pole from active neovascularization with inadequate laser treatment. Bottom image (**B**) shows quiescent clinical disease following vitrectomy and endophotocoagulation. Visual acuity improved from count fingers to 20/40. (Images courtesy of Siva S.R. Iyer, MD).

The VEGF family consists of seven proteins, with VEGF-A being the one most studied and pathogenic in PDR [69,76,77]. The source of VEGF is endothelial cells and the retinal pigment epithelium [78]. VEGF-A, in response to hypoxic environments, is upregulated and binds to the endothelial-expressed VEGF receptor 2 and stimulates angiogenesis and vascular permeability, resulting in proliferative diabetic retinopathy [76,79]. Aberrant neovascularization is prone to leakage and hemorrhaging that are the hallmarks of advanced active PDR (Figure 2).

VEGF promotes endothelial permeability by reducing the quantity of occludins at tight junctions and activating protein kinase C beta (PKC-β) [80,81]. It is well established that VEGF is elevated in the vitreous humor of patients with PDR as well as diabetic macular edema (DME) and is an important therapeutic target [16,22,24,35,37,41,52,79,82,83,84,85,86]. Perrin et al. demonstrated that one isoform of VEGF, VEGFxxxb, which is antiangiogenic in nature, is downregulated in the vitreous humor in patients with PDR. In normal eyes, VEGFxxxb consists of ⅔ of the VEGF found in the vitreous humor, acting to inhibit vessel growth. However, in patients with PDR, it is significantly decreased compared with the angiogenic form of VEGF [87].

VEGF has also been detected in other conditions such as rhegmatogenous retinal detachment, albeit generally at lower levels [37]. In NPDR, some animal studies have shown increases in VEGF [50] while other clinical studies have shown a normal range [51] or even higher levels of VEGF [52]. In PDR, VEGF serum levels do not correlate with vitreous concentrations, with serum concentrations being comparable to non-DR and non-diabetic eyes [22]. With a lack of a serum correlation, VEGF generation is likely primarily local [22]. VEGF concentrations also do not appear to correlate with serum hemoglobin A1c levels [88,89]. This may be due to glycemic control prior to vitrectomy in studies and not necessarily reflective of the DR severity and subsequent vitreous composition in NPDR and PDR [89,90]. Further analysis such as plasma protein analysis and protein normalization studies also argue against a VEGF spill from the serum into the vitreous with blood–eye breakdown that is inherent in DR [22,29].

Placenta Growth Factor (PlGF) is a member of the VEGF family [73]. It is thought to act indirectly and directly through the VEGF receptors, such as VEGF-R1, to upregulate angiogenesis [91]. PIGF is reported to stimulate subretinal fluid accumulation and the formation of fibrovascular membranes that are associated with PDR [92,93]. It has also been reported to be elevated in the PDR vitreous humor [73,76]. Furthermore, animal studies have shown that deletion of the PIGF gene prevents VEGF-A activation [94]. Thus, PIGF is an important regulator of angiogenesis demonstrating the complex pathogenesis behind PDR.

PDGF is a cell maturation and potent angiogenic growth factor that affects the development and survival of cells [95,96]. PDGF is released mainly from alpha granules of platelets [97]. The B chain, PDGF-B, has been implicated in retinal pericyte health [96]. PDGF shares structural homology with VEGF and has been implicated in the pathogenesis of PDR [95,96]. PDGF has been shown to be elevated in the human PDR vitreous in multiple studies [34,70,95]. PDGF (PDGF-A, and PDGF-B and homo-/heterodimer forms) correlates with levels of VEGF in the PDR vitreous, and its levels are not significantly different in serum compared to controls [88,90]. Similar to VEGF, this implies a local source for this growth factor [90]. In fact, PDGF levels were shown to be lower in the PDR vitreous of eyes that underwent PRP prior to vitrectomy compared to eyes that did not undergo preoperative PRP [34,88]. PDGF has not, however, been shown to correlate with the presence of a fibrovascular membrane [88].

Basic fibroblast growth factor (FGF-2) is a strong pro-angiogenic factor and mitogen for endothelial cells [98]. FGF-2 acts through both the extracellular signal-regulated kinase (ERK1/2) and the phosphoinositide 3 kinase (PI-3K) pathways depending on the cell type [98]. FGF-2 may also be synergistically enhanced when combined with other specific growth factors. For example, when combined with PIGF, FGF-2 stimulated the proliferation of bovine endothelial cells, whereas FGF-2’s effects were minimal when acting alone [99]. FGF-2’s pro-angiogenic effects have also been demonstrated in bovine retinal endothelial cell secondary sprouting assays [99]. Treatment with intravitreal bevacizumab reduces vitreous FGF-2 levels in the diabetic eye with subsequent effects on retinal thickness, suggesting a local role for FGF-2 [100]. FGF-2 is thought to blunt some of the vitreous-stimulated morphological changes to RPE cells [101]. In DR, FGF-2 is found in the basement membranes of blood vessels; however, as DR severity increases, FGF-2 within the retina increases as well. With conversion to PDR, FGF-2 is found in an intracellular location rather than in the basement membrane [102]. FGF-2 may also have a role in inducing VEGF and PDGF [103].

TGFβ is a proliferative growth factor that can induce action through many pathways including pro-fibrotic cell proliferation and migration both independently and via connective tissue growth factor (CTGF) in the eye [72,104]. TGF-β is released from the alpha granules in platelets, activated T-lymphocytes, and macrophages, the most likely source in diabetic retinopathy [105,106,107,108,109]. The TGF-β_2_ isoform has been shown to be elevated in the PDR vitreous at two–three times the level compared to controls [72]. In the first study showing elevated levels of TGF-β2 in the vitreous in PDR, insulin-treated patients showed higher levels within PDR eyes [110]. Its effects on extracellular matrix aggregation likely add to the deleterious effects of aberrant vessel formation to help create fibrovascular membranes in PDR [72]. Independent of TGFβ, CTGF has been specifically studied in the vitreous of active PDR eyes, and the accumulation of the NH_2_-terminal CTGF fragment (insulin like growth factor domain and von Willebrand type C domains) was at the highest levels compared to inactive PDR and non-diabetic controls [111]. The stable fragment may represent a marker of CTGF activity in active PDR following proteolysis from enzymes such as metalloproteases (MMPs) [111]. CTGF shares a balance with VEGF in the change from pro-angiogenic to pro-fibrotic action that is influential in the formation of TRD [112].

Not only does the current literature report evidence of pro-angiogenic and pro-inflammatory modulators in the vitreous humor, but there is also support for a decrease in anti-inflammatory modulators as well [42,86]. Pigment epithelial derived growth factor (PEDF) is seen in decreasing quantities in patients with proliferative diabetic retinopathy when compared to those without [54,84]. PEDF is an anti-angiogenic growth factor that acts to thwart endothelial cell migration as well as proliferation [113]. PEDF is secreted by the RPE and ciliary epithelium [114,115]. Although one study [113] found elevated levels of PEDF in the PDR vitreous, many studies have consistently shown decreased levels of this cytokine in the PDR vitreous [41,42,86,116,117].

PDR pathogenesis proceeds via three main and related pathways: angiogenic, inflammatory, and fibrotic [13,68,112,113]. This understanding of the changes in the vitreous humor has led to the mainstay treatments for PDR, namely, anti-VEGF. However, the frequent recurrence and resistance of disease, despite this treatment, are some of the underlying reasons why elucidation of alternative pathways to VEGF in disease promotion is critical. It is now established that VEGF-independent pathways are important in DR pathogenesis [84,113,118]. These pathways contribute to the severity of PDR and play a role in resistance to therapy, as commonly seen in refractory diabetic macular edema [83,119].

There is now ample evidence supporting the role of inflammation in the pathogenesis of proliferative diabetic retinopathy [82,120]. Pro-inflammatory cytokines IL-6, IL-8, TNF-α, monocyte chemoattractant protein-1 (MCP-1), and macrophage inflammatory protein-1β (MIP-1β) have all been reported to be elevated in the diabetic vitreous [34,54,113,120]. Cumulative data over several studies in the DR vitreous show a consistent group of prominent chemokines and cytokines that have emerged (Table 1). The next section reviews those mediators specific to PDR human vitreous studies.

### 3.5. Interleukins

Interleukins are a group of inflammatory cytokines important in the regulation of immunogenic responses to disease or trauma and have been characterized in the diabetic vitreous in several studies [24,25,41,83,124,133,137,139,140,141]. Analytical results have been mixed regarding the vitreous and serum correlation with different cytokine levels [127,138,142]. Interleukin-6 and interleukin-8 vitreous levels have been shown to be consistently elevated in PDR eyes [24,27,41,132,138,142,143]. Koskela et al. showed that IL-6 and IL-8 levels in the vitreous were significantly elevated vs. serum in PDR, and also elevated compared to non-diabetic controls, suggestive of a local role for both of these inflammatory cytokines in PDR [132].

Interleukin-6 (IL-6) is a pleiotropic inflammatory cytokine with a multitude of functions including the enhancement of leukocyte accumulation, and macrophage activation [125,141]. IL-6 is also a pro-angiogenic cytokine [144]. IL-6 levels have been measured in the PDR vitreous and found to be consistently elevated [24,27,34,54,121,125]. Although it is elevated in the diabetic vitreous, much like many of the other growth factors and inflammatory mediators, it is not simultaneously elevated in the serum across many studies [27,35,41,54,84,124,126]. IL-6 production can be induced by advanced glycation end products [126]. In the ischemic diabetic retina, IL-6 is thought to act upstream of VEGF to induce its expression and even act on endothelial cells to cause increased permeability [35,79,118,141]. Inhibition with an IL-6 neutralizing antibody added to the human PDR vitreous has been shown to have a blunting effect on capillary tube formation in vitro [24]. Despite these direct and indirect effects of IL-6, it has been found to be elevated in non-PDR eyes and may not be a specific marker necessarily of PDR or DR in general [133]. In fact, IL-6 levels were higher in PDR eyes that underwent pan-retinal photocoagulation (PRP) one week prior to vitrectomy compared to contralateral eyes that did not undergo PRP independent of VEGF levels [31,32]. The elevated levels of IL-6 in treated PDR eyes may represent more than one active pathway in the evolution of PDR.

Interleukin-8 (IL-8) is a hypoxia-induced chemoattractant from endothelial and glial cells that activates neutrophils and T lymphocytes [64,120]. It is regarded as a pro-angiogenic chemokine [139]. Its action is mediated by binding of the transcription factor nuclear factor kappa-B (NF-κB), which regulates several genes that affect pro-angiogenesis and adhesion mediators [64]. IL-8 has a role in angiogenesis and vascular integrity loss in DR [130]. When the vitreous of active PDR was compared to inactive PDR in an early study of IL-8, there was no difference between groups [41]; however, subsequent studies of IL-8 levels have consistently shown to be from 2 times to nearly 100 times higher in active PDR than controls, and even 10 times higher in quiescent PDR compared to controls [24,34,35,113,120,122,130]. IL-8 has been found in retinal endothelial and glial cells by immunohistochemistry, suggesting a source [64]. A direct correlation of IL-8 levels with VEGF has been demonstrated in the PDR vitreous [18], and like IL-6, it has not been found to be significantly elevated in the serum of PDR patients [41,125]. In PDR eyes that have undergone pan-retinal photocoagulation (PRP) prior to vitrectomy, IL-8 vitreous levels have been shown to be to lower than in PDR eyes that had not undergone laser treatment [34]. VEGF levels interestingly show little difference in laser preoperative eyes compared to intraoperative lasered eyes when the two have been compared [30]. In the vitrectomized eye, IL-8 levels in the posterior segment have been shown to decrease significantly compared to their pre-vitrectomized levels [134]. IL-8 has also been shown in a univariate analysis to negatively correspond with post-surgical visual acuity levels along with macular attachment and preoperative photocoagulation status [128].

Interleukin-1-beta (IL1-β), another pro-inflammatory cytokine, is secreted in a pre-processed form (pro-IL1-β) and then undergoes cleavage by caspase-1 to release its active form [135,145]. The inhibition of caspase-1 and the active form of IL-1β has been shown to blunt the downstream deleterious effects of these mediators on capillary health in a mouse model [145]. In contrast, the stimulation of caspase-1 by inflammasomes such as the NOD-like receptor family, pyrin-domain containing 3 (NLRP3) can lead to active IL1-β and IL-18 [146]. These findings suggest this signaling process is vital to the pathogenesis of retinal ischemia and further hypoxia-induced retinal neovascularization. NLRP3 may be upregulated by ATP release from the extracellular space or from reactive oxygen species [146]. Yet, IL1-β analysis in the PDR human vitreous has revealed inconclusive results, with some studies showing elevated levels and other studies showing little to no change [24,40,43,127,135,147]. Perhaps accurate measurement of IL1-B in the vitreous may be difficult due to its short half-life [43]. Zhao et al. evaluated a limited number of PDR eyes for IL-1β and pro-IL1-β in the vitreous and found no significant differences between PDR eyes vs. controls (epiretinal membranes) [135]. The authors commented that their lower levels may be the result of less severe PDR as their sampling contained only VH and no TRD eyes [135]. IL1-β measured in another study of PDR vs. NPDR patients showed elevation in PDR eyes but not NPDR eyes vs. controls [129]. Yet, another study found nominally elevated levels in DR and not in all samples tested [147]. Limited studies have found significantly elevated levels in the PDR vitreous along with serum levels that were greater in PDR eyes compared to controls, suggesting local production in macrophages and the retinal pigment epithelium [127]. Taken as a whole, the studies on IL1-β support the notion that this cytokine is likely produced locally and increases in concentration with DR severity but may not play as prominent a role as IL-6 and IL-8 in DR pathogenesis.

IL-10 (cytokine synthesis inhibitory factor), an anti-inflammatory cytokine, has been shown to be elevated, decreased, and also unchanged in the vitreous in PDR [24,40,43,113,120,131,132,133,138,143,148]. The reason for this is not entirely clear but may be partially due to varying sampling methods and patient co-morbidities that could alter the immune system [133]. In one study, IL-10 vitreous levels were significantly lower than the serum measured levels [132]. In this example, higher IL-6 may have had an influence on upregulating the IL-10 levels measured [132]. Another study showed elevated levels of pro-inflammatory cytokines such as IL-9 and MCP-1 were not associated with elevated levels of IL-10 in the same vitreous sample [120]. This suggests that IL-10 levels are not necessarily higher to counter-effect the pro-inflammatory response. Tan et al. conducted a meta-analysis with inclusion of eleven studies showing increased IL-10 levels in the vitreous rising to significance but not in plasma or serum [148]. Due to the uncertain meaning of IL-10 levels at this time, this area requires further research. As of now, IL-10 appears unlikely to play a key role in PDR pathogenesis.

T-helper 17 (Th17) cells are involved in the production of IL-17A, an inflammatory cytokine with a role in autoimmunity and downstream production of cytokines such as TNF-α [133,136]. Th17 production is thought to be downregulated by IL-35 expression (produced by T and B lymphocytes) [25]. Interestingly, it has been shown that IL-17A and Th17 levels were increased in the diabetic (non-DR) peripheral circulation with decreasing levels with more advanced DR [136]. The vitreous concentration of IL-17A was shown to be increased in PDR [133,136]. Taken together, this suggests that the blood–retinal barrier breakdown may allow decreasing levels in the circulation and an increase in vitreous levels as DR progresses. IL-17A has also been shown to correlate with TNF-α levels in the PDR vitreous which supports this notion [133]. IL-35, in contrast, has been shown to be at significantly lower levels in the serum and vitreous of PDR eyes compared to controls. In NPDR eyes, however, the levels were comparable to controls [25]. This highlights a possible autoimmune role for elevated interleukin levels with downstream independent cytokine effects in DR that may vary with the severity of DR.

Other lesser known members of the interleukin family have been minimally investigated in DR. Interleukin-26 (IL-26), a pro-inflammatory cytokine, has been reported to be elevated in the vitreous and serum of patients with PDR [137]. The protein concentration of IL-26 in the vitreous is also inhibited by anti-VEGF therapy as eyes treated with anti-VEGF had significantly less levels than those that had not been treated [137]. IL-26 mRNA has also been detected at higher levels in peripheral blood mononuclear cells from PDR patients [137]. IL-27 has not been well investigated in PDR but is thought to have multiple effects on immune regulation with the stimulation of T-regulatory cells (that secrete IL-10) and promote tissue healing [25]. It has been shown, along with IL-35 (an anti-inflammatory cytokine that inhibits T cell production), to be lower in the serum of PDR eyes vs. controls (and NPDR) [25]. IL-27 was neither significantly higher or lower in the PDR vitreous compared to controls [25]. IL-31 was shown to be elevated in the PDR serum and vitreous compared to controls in one study [133]. IL-31’s role in PDR is not known at this time. IL-12 is anti-angiogenic and was found to be lower in the PDR vitreous compared to non-diabetic controls, and lower in the vitreous compared to the serum [43]. IL-12 (and IFN-γ) levels in the PDR vitreous are shown to decrease after bevacizumab treatment [149]. Finally, IL-37 has been specifically shown in one study to be elevated in the PDR vitreous and correlated with VEGF levels, with stimulation of endothelial cells in vitro in an angiogenic role [150].

### 3.6. Major Cytokines

Monocyte chemoattractant protein-1 (MCP-1), also known as chemokine (C-C) ligand 2 (CCL2) [120], exhibits chemoattractant properties for monocytes and T lymphocytes [120]. These two cytokines are increased in the diabetic vitreous and are mediators of PDR pathogenesis [21,24,54,113,120,122]. Together, IL-8 and MCP-1 recruit inflammatory cells which in turn secrete angiogenic factors [130]. MCP-1 levels can be induced by TNF-α and interferon-γ [123]. MCP-1 levels have been found to be seven times the level in PDR vs. control eyes and correlate with simultaneously measured VEGF levels [34,134]. MCP-1 levels and IL-8 levels show a correlation with each other in the vitreous and also in fibrovascular membranes in PDR [40,43]. MCP-1 also showed a correlation with IL-6 levels [123]. In one of the earliest vitreous studies of MCP-1, levels were found to be elevated in PDR, undetectable in control eyes with epiretinal membranes, and undetectable in the corresponding PDR serum [123]. The many corroborating studies mentioned collectively show that MCP-1 has a strong role in the pathogenesis of PDR by recruitment of cells in response to injury leading to angiogenic and fibrotic change.

Intercellular adhesion molecule-1 (ICAM-1) is directly involved in the process of vascular permeability leading to a leukocytosis in the retina [84,151,152]. ICAM-1 is expressed by the vascular endothelium and is upregulated in patients with diabetes in response to systemic TNF-α [152]. Leukocytes bind to ICAM-1 in the extravasation process, enter the retina, and subsequently release more pro-inflammatory growth factors and cytokines [84,152]. ICAM-1 is also involved in the enhancement of VEGF [84]. In mice models where ICAM-1 was deleted, VEGF-induced leukostasis and blood–retina barrier breakdown were decreased [151]. ICAM levels are elevated in the PDR vitreous, without concurrent serum level elevation [33,153]. Soluble intercellular adhesion molecule-1 (sICAM 1), soluble platelet-endothelial cell adhesion molecule (sPECAM-1), selectin, and soluble vascular cell adhesion molecule (sVCAM-1) in multiple studies have been shown to be elevated in the vitreous of PDR eyes but not significantly different than their counterpart serum levels [33,132,138]. Overall, these results favor a more localized role for interleukins and a systemic stimulation of adhesion molecules.

Stromal cell derived factor-1 (SDF-1) is a chemokine primarily produced by bone marrow stromal cells that is known to stimulate hematopoietic cells and endothelial progenitor cells in response to tissue injury [154,155]. SDF-1 mediates its effects through the G protein-coupled receptor CXCR-4, which allows hematopoietic cells to transmigrate through the bone marrow endothelium [155]. Furthermore, the literature reports its upregulation in other disease states that not only affect the eye, but the liver and brain as well [154,156,157]. However, the primary mechanism of action of SDF-1 in the diabetic vitreous is thought to be through the upregulation of VCAM-1 and downregulation of occludins, which facilitates endothelial progenitor cell mobilization to the site of retinal injury. SDF-1 induces the expression of VCAM-1 on retinal endothelial cells which allows the migration of endothelial progenitor cells from the bone marrow [154]. Butler et al. established a murine model that when SDF-1 was introduced, it had similar effects on retinal neovascularization to VEGF exogenously [154]. SDF-1 has been detected at elevated levels in the PDR and DME vitreous [158]. An additive effect exists between SDF-1 and VEGF, with SDF-1 stimulating VEGF and VEGF making cells more susceptible to the effects of SDF-1 [158].

Tumor necrosis factor-alpha (TNF-α) is a main pro-inflammatory cytokine and has been investigated in the diabetic vitreous in many studies [34,121,125]. It has been reported to be higher in serum in DR but not in the vitreous [38,125], but it has also been reported to be higher in both [33,34,39]. TNF-α, similar to IL-1β, may be difficult to detect in the vitreous because of its short half-life [43]. Due to conflicting data [35,43], and although TNF-α has been shown in cells from PDR fibrovascular membranes [159], the source of TNF-α is not well understood at this time. TNF-α, however, is also higher in the vitreous with PDR vs. without retinopathy, suggestive of some element of local production in active disease.

Matrix metalloproteases are enzymes that are secreted in their inactive form and are activated depending on the conditions of their environment [160]. They have key roles in the remodeling of the extracellular matrix [160]. Previous studies have also shown elevated levels of matrix metalloprotease-2 (to a lesser degree) and 9 (MMP-2 and MMP-9) in the diabetic vitreous as compared to controls [65,160,161,162]. The cellular content of fibrovascular membranes may harbor MMP-producing cell types including RPE fibroblasts and glial cells [160]. The active form of MMP-9 (activated by MMP-3) may be produced within PDR micro-vessels and released into the vitreous. Elevated levels of tissue inhibitor of metalloprotease production (TIMP) within the vitreous of PDR eyes also support this local production and release of MMP-9 [161].

IFN-γ, part of the helper-T group of response, is part of the innate inflammatory immunity. It has been shown to be elevated in DR patients, but no significant correlation between PDR and NPDR could be established in one small study [91].

**Figure 3:**
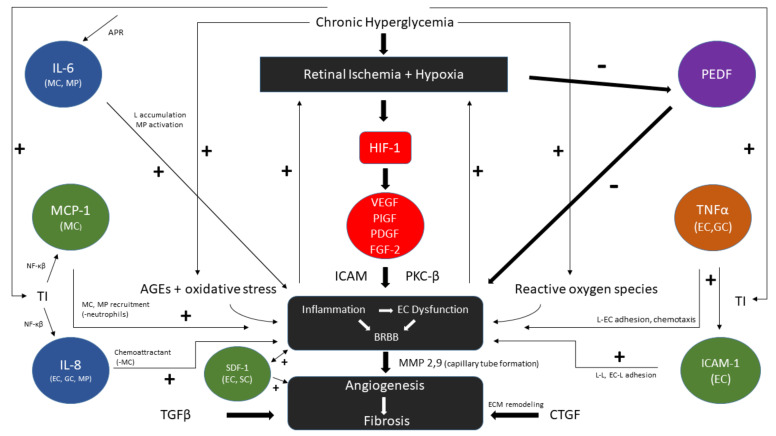
**Flow diagram of VEGF dependent and major independent patho-mechanisms in proliferative diabetic retinopathy.** Chronic hyperglycemia leads to tissue injury (TI), hypoxia, advanced glycated end products (AGEs), and reactive oxygen species that cause inflammation and endothelial dysfunction with blood–retinal barrier breakdown (BRBB) in a positive feedback loop. VEGF is produced directly by HIF-1 stimulation in hypoxic conditions and directs its action through PKC-β and stimulates ICAM. PEDF and its inhibitory effects on BRBB are suppressed by hypoxia. VEGF independent pathways include IL-6, by acute phase response (APR) with induction of leukocyte (L) accumulation. TI further stimulates MCP-1 and IL-8 through NF-κβ as chemoattractants. TNFα secreted by endothelial cells (EC) and glial cells (GC) and ICAM-1 promote EC and L adhesion to further cause loss of microvascular integrity. TNF-α stimulates MCP-1 (not shown). SDF-1, derived from EC and stromal cells (SC) (bone marrow), is generated in response to endothelial injury and recruits progenitor cells, weakens tight junctions, and may promote neovascularization. Continued microangiopathy results in angiogenesis and culminates in fibrotic extracellular matrix (ECM) remodeling (through TGFβ, CTGF) whose footprint is largely irreversible. The above concepts have been discussed throughout the ongoing PDR vitreous scientific literature [21,35,40,42,64,81,94,99,112,120,123,134,154,163,164,165].

When the diabetic vitreous literature is examined collectively, we can begin to piece together a possible flow of events leading to mechanical retinal injury (Figure 3).

### 3.7. Other Mediators

Most of the major cytokines and chemokines involved in PDR have been discussed above. There are other single or small study reports that have investigated novel proteins and other signal cascade members and processes. We have mentioned a few below.

Natural polyamines, essential for cell growth and development, have been studied in the diabetic vitreous in only one study [69], and spermindine and spermine were shown to be elevated in the PDR vitreous [69]. This was true for both active PDR and quiescent PDR compared to controls. Spermine was found to be specific to PDR and absent in controls, suggesting that it may be a marker for angiogenic diabetic retinal disease [69]. Putrescine, a precursor to spermidine, was shown to be decreased in the PDR vitreous compared to controls [69]. Heperanase, key to the metabolism of heparin sulfate, has been implicated in pro-angiogenic and inflammatory processes. Heparin sulfate proteoglycan 2 has shown an over 11-fold increase in the PDR vitreous [53]. Heperanase in the PDR vitreous in one study was significantly elevated vs. controls (rhegmatogenous retinal detachment) [36]. The heparanase protein was found in the fibrovascular membrane and vascular endothelial cells of these same eyes, supporting a local production source and involvement in PDR advancement [36].

Adiponectin is an anti-angiogenic protein with protective effects against diabetes and diabetic damage [65]. Decreased levels of adiponectin in the vitreous have been shown in both rodent and human models [65]. This protective role is not common among most proteins studied and may be a future promotional target. Hydrogen sulfide (H2S), a neuromodulatory gas that can migrate across cell barriers without assistance, has been shown to be elevated in the vitreous of PDR eyes, and in the serum of DR patients, with a decrease in the serum from patients without DR [28]. Homocysteine in DR patients and retinal ischemia with VEGF production may stimulate H2S, which can lead to endothelial cell replication and activity. This notion was further supported as H2S vitreous levels decreased after ranibizumab treatment [28].

Interestingly, RNA can play a role in the patho-mechanism of microvascular destruction. The circular RNA cZNF532 has been shown to be upregulated in eyes with DR, and this higher expression has been shown with elevated levels in the vitreous of patients with DR; these levels correlate with diabetic retinopathy severity [48]. cZNF532 serves a protective role that inhibits miR-29a-3p (microRNA) to protect the retina from pericyte apoptosis [48]. Newer studies may open the door to potential non-conventional therapeutic targets such as nucleic acids.

### 3.8. Correlation to VEGF Levels

As noted above, VEGF intravitreal levels in the PDR vitreous have consistently been shown to be elevated compared to less severe DR and controls, but without a correlation to serum levels [29]. This trend is also true for IL-6, IL-8, and MCP-1, as extensively discussed above [24,27,41,84,123,124,125].

IL-6, IL-8, and MCP-1 levels correlate well with measured VEGF levels [41,84,166]. Furthermore, treatment with bevacizumab does not show a reduction in these pro-inflammatory cytokines, further supporting the notion that these mediators work in a VEGF-independent fashion [149]. IL-10 and IL-13 have also been linked to increased VEGF levels in PDR eyes [131]. A correlation has been shown between elevated pentosidine (an advanced glycation end product) in the PDR vitreous and VEGF [126,167].

The coagulation pathways and angiogenic pathways may have a significant relationship in PDR pathogenesis, the specifics of which are largely unknown. Thrombin and matrix metalloprotease-1 (MMP-1, a pro-angiogenic collagenase) and thrombin have both been associated with increased VEGF vitreous levels in PDR [37]. In a 1999 study, tissue plasminogen activator (tPA) and plasminogen activator inhibitor were shown to be elevated in the PDR vs. the non-diabetic vitreous [85]. Thrombin is known to stimulate IL-6 production [168]. Thrombin–antithrombin (TAT) complex and D-dimer levels are also elevated in the PDR vitreous [166,168]. A correlation was found between D-dimer and IL-8 as well [166]. Given IL-6’s and IL-8’s documented correlation to VEGF levels, there may be cross-talk at this level between the two systems. Interestingly, there has not been an established correlation between vitreous TAT levels and IL-6, or VEGF, in DR [168], indicating that these two systems may communicate, but possibly work, at least partly, independently [166,168].

### 3.9. Active vs. Inactive PDR

VEGF levels in active vs. quiescent PDR show mixed results. Some studies show VEGF persistently elevated even in the quiescent state (although it may be lower than in active PDR) [22,29,169], while others show it to be non-significant from controls [69]. This variability may arise from the ill-defined “inactive or quiescent” state (clinical observation, angiographic evidence, etc.) [18,170,171]. Wang et al. showed nearly twice the VEGF concentration in active vs. quiescent PDR and 1.3 times with intravitreal VEGF/total protein adjustment [29]. This consistency did not hold true for hypoxia inducible factor 1 (HIF-1) after protein concentration adjustment. Given that HIF-1 is an upstream inducer of VEGF, the lack of consistent elevation suggests a baseline level of VEGF in the PDR eye [29].

PEDF has been shown to be lower in active PDR compared to inactive PDR and lower in eyes with PDR vs. NPDR [116]. This is in line with the general understanding that active PDR and PDR (vs. NDRP) represent higher angiogenic states and would likely have less anti-angiogenic activity. Insulin like growth factor 1 (IGF-1) is elevated in the PDR vitreous and has been shown to be significantly elevated in all forms of PDR compared to non-diabetic eyes [110]. TGF-β2, IGF-1, and basic fibroblast growth factor (bFGF) have all been detected at higher levels in active PDR vs. quiescent PDR in the vitreous [67,110]. This supports the view that local pro-angiogenic and fibrotic growth factor levels are necessary to induce and maintain active disease.

Other lesser studied members of the interleukin family have roles in active PDR. IL-18, another multifunctional cytokine, and inducer of IL-6 and IL-8, was evaluated in the PDR vitreous in the active vs. inactive states and found to strongly correlate with VEGF in only the active PDR state, although levels of IL-18 and VEGF were both higher across all PDR eyes vs. non-diabetic controls [169]. IL-37, a newer member of the IL-1 family and thought to act through the action of IL-18, has been shown in one study to be elevated in the PDR vitreous and correlate with VEGF-A and angiopoietin-2 (Ang-2) levels [150]. The authors of this study further demonstrated that IL-37 stimulates VEGF-A and chorioretinal endothelial tube formation and cell proliferation [150].

Over 15 years ago, Funatsu et al. showed that angiotensin II levels were higher along with VEGF in the vitreous and serum of PDR patients in the active stage of PDR [172]. Although angiotensin II was significantly higher in the vitreous compared to serum, the margin was small. The rise in vitreous angiotensin II levels may be due, in part, to its migration following a collapse of the blood–retinal barrier with active PDR [172].

**Figure 4:**
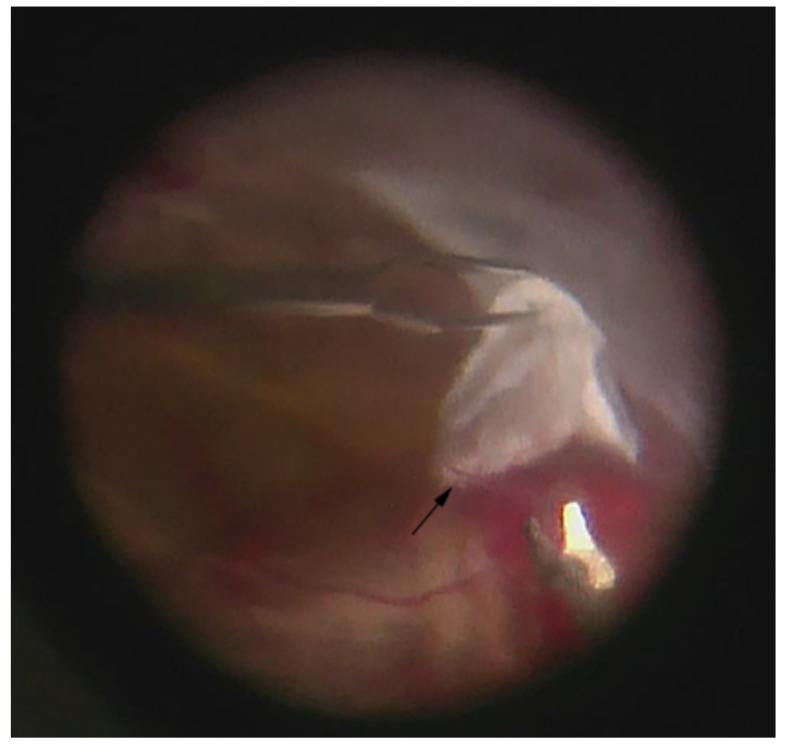
**Fibrovascular membrane of a tractional retinal detachment (TRD).** A combination of forceps and scissors is used to delaminate and remove the membrane from the retinal surface without injury to the retina. Remnant neovascularization (arrow) can be seen at the base of the white fibrous membrane as the majority has regressed following preoperative treatment with bevacizumab. (Intraoperative image courtesy of Siva S.R. Iyer, MD).

As a whole, the majority of the literature that has studied the vitreous in PDR shows an expected elevated change in the concentration of major and some minor cytokines in active PDR. It is clear that cytokines that play a role in diabetic retinopathy are produced from retinal cells, the retinal pigment epithelium, pericytes, endothelial cells, Müller cells, astrocytes, and hyalocytes [173]. The pathogenesis of diabetic retinopathy cannot be summarized only in the retina. The vitreous can act as a reservoir for these mediators, and this is the reason that eyes that undergo a vitrectomy have long-lasting benefits [174]. The vitreous plays more of a role in the proliferative stage and in membrane formation. Fibrovascular membranes are composed of many cell types, i.e., hyalocytes, fibroblasts, retinal glial cells, macrophages/monocytes, laminocytes, and vascular endothelial cells [175]. The hyalocyte cells which are a component of the vitreous can be a source for connective tissue growth factor (CTGF) and may play a role in the formation of fibrous tissue in the proliferative stage. Hyalocytes not only have a role in the proliferative stage but also in immunological disorders if we consider diabetic retinopathy an autoimmune process. Hyalocytes may have a role for changes in the vitreous body as a source of antibodies [175]. As active PDR changes to early TRD formation (Figure 4), an increase in vitreous pro-fibrotic factors and cytokines within cell types (e.g., stromal, endothelial) in fibrovascular vascular membranes has been shown in multiple studies [36,43,112,171,176].

### 3.10. Complement

Even prior to the clinical evidence of DR, the pro-inflammatory and neuro-degenerative pathways that start damage early in the diabetic retina may be immune-mediated and involve the complement system [49,56]. The complement system consists of three major pathways: classic, alternative, and lectin [177]. Complement in the diabetic vitreous has been investigated in recent years [138,178,179]. The classic pathway has not been implied in vitreous studies in PDR eyes. The alternative pathway, however, with elevated levels of the central complement C3 and specifically C3bα′, C5a, and Ba, has been noted in the vitreous of PDR eyes [53,138,178,179]. Similar to the interpretation of elevated interleukins, the question of whether circulating complement may have influenced vitreous levels is important. For example, lipopolysaccharide (LPS), through passage from the breakdown of the blood–eye barrier, may be involved in activating the complement system in PDR [26]. However, in one recent study, elevated levels of complement suggesting activation of the alternative pathway were confirmed with correction for vascular leakage in the vitreous (usage of the albumin ratio [53]) to exclude influences from the general circulation [179]. A fragment of C5, C5a, has been shown to be elevated in the PDR vitreous to levels 16 times control levels, without a concomitant increase in serum, also suggesting production from local tissues [178]. C5a has also been implied to stimulate production of pro-inflammatory cytokines such as IL-8 from retinal pigment epithelial cells but that these levels did not correlate quantitatively, implicating that downstream stimulation of proteomic mediators is not easily predictable but likely involves a more complex system [178]. Complement factor H (CFH), a downregulator of the alternative complement pathway, was shown to be elevated in the PDR vitreous but not in the corresponding serum, suggesting secretion from the retina during advanced DR [138]. The complement and coagulation pathways are linked together in the pathogenesis of PDR, but the specifics of this relationship are not well understood. The components used in the coagulation system, as well as those in the kinin–kallikrien system, are upregulated in the conversion of NPDR to PDR [56].

### 3.11. Diabetic Macular Edema

In human eyes with non-proliferative diabetic retinopathy with diabetic macular edema (DME), the vitreous levels of VEGF have been measured to be more than 50 times the level in non-diabetic eyes and more than 30 times the level in diabetic eyes without retinopathy [84]. The vitreous level of VEGF has been shown to be over 20 times the levels in plasma from the same patients with DME [42]. VEGF has been found to follow a gradient in DME eyes, with a higher concentration in the pre-macular area compared to the mid-vitreous and cortical vitreous in the periphery [23]. The amount of VEGF (regardless of the location where it was measured) correlated with central macular thickness [23]. PEDF, an anti-angiogenic glycoprotein, levels were lower in DME eyes, with corresponding lower values to increasing retinal thickening [42,84]. The amount of leakage on preoperative fluorescein angiography also correlated with increased levels of VEGF, but a deceased level of PEDF, indicating that a compromise of the blood–retinal barrier plays a role in the release of hypoxic and inflammatory cytokines into the vitreous [84,180]. In the post-vitrectomized eye, DME was most highly correlated with MCP-1 levels, indicating that MCP-1 may play an important role in post-vitrectomized inflammation leading to persistent DME [134].

The IL-1β vitreous concentration was found to be 2-fold higher and interleukin 1 receptor antagonist (IL-1RA) 57-fold higher than non-diabetic controls in DME eyes [17]. The IL-1RA/IL-1β ratio was found to be 13-fold higher and may play a role in the delicate inflammatory balance in the development of DME [17].

IL-6 is expectedly involved in the pathogenesis of DME, as shown in the literature, with greater levels found in DME eyes [27,170]. It has been shown to be specifically related to subretinal fluid in DME by a possible contribution to external limiting membrane disruption, allowing migration of fluid into the subretinal space [170]. In eyes that underwent PRP prior to vitrectomy, IL-6 and Regulated upon Activation, Normal T Cell Expressed and Presumably Secreted (RANTES) levels increased compared to contralateral eyes that did not undergo pre-surgical PRP and correlated directly with DME, but not with VEGF levels [31,32]. Temporary induction of capillary permeability following scatter laser treatment may follow a VEGF-independent pathway.

Endothelin-1 (ET-1), an endothelial cell-derived peptide, was found to have a positive vitreous level correlation in eyes with non-proliferative DME [129]. This same study showed decreased amounts in NPDR eyes compared to both PDR eyes and controls (idiopathic macular holes) [129]. It is perhaps that the receptor-mediated vasoconstrictive effects of ET-1 are more prevalent in PDR, whereas in NPDR with DME, a receptor-mediated vasodilatory effect predominates [129]. ET-1 has been shown, however, to be elevated in the PDR vitreous and correlate with the HgA1C concentration [39,40]. Due to mixed results of ET-1 in vitreous profiles with diabetic severity, this area needs further data to be conclusive.

ICAM-1 levels were at least twice the levels compared to non-diabetic and eyes without retinopathy. Levels of MCP-1 were four times higher compared to non-diabetic eyes and two times higher than diabetic eyes without retinopathy [84]. When less severe DME (minimally fluorescent) was compared to more severe DME (hyperfluorescent), levels of MCP-1 approached twice the level in the vitreous [84]. VEGF and ICAM-1 levels were more associated with the severity of DME compared to others. ICAM-1 was also correlated with the severity of diabetic macular edema in a 2009 study by Funatsu et al. [84].

### 3.12. Lipid-Derived Signaling Molecules

Tight glycemic control is one of the main preventative factors expressed in patients with diabetes. However, tight glycemic control does not fully eliminate the risk of developing diabetic retinopathy, thus underscoring the importance of understanding the diabetic milieu, which includes dyslipidemia [181,182].

Polyunsaturated fatty acids are important mediators in the pathogenesis of proliferative diabetic retinopathy, namely, arachidonic acids and its derivatives. Currently, derivatives such as eicosanoids, dihydroxyeicosatraenoic acid (DiHETEs), leukotrienes, and epoxyeicosatrienoic acid have been investigated as retinal angiogenic mediators [13]. Arachidonic acid pathways contributing to the diabetic vitreous environment include the cyclooxygenase (COX), lipoxygenase (LOX), and CYP 450 pathways [13,34,183,184,185,186,187].

The cyclooxygnase enzyme pathway converts polyunsaturated fatty acids into prostaglandins from arachidonic acid. These bioactive lipids can disrupt the blood–retinal barrier, increase vasodilation, and induce leukocyte migration [34,183]. PGE2 levels specifically are reported to be increased in the PDR vitreous [34]. Schoenberger et al. reported a correlation with increased levels of PGE2 and VEGF. This further supports the inflammatory nature of diabetic retinopathy [34].

The lipoxygenase pathway converts arachidonic acid into 5-HETE (leukotriene pathway), 12-HETE, and 15-HETE [13,188]. 5-HETE is reported to be increased in the PDR environment [3]. Although not previously shown to be a strong inducer of inflammation, it may be a marker for increased 5-LOX activity as well as a precursor to pro-inflammatory leukotrienes [13,189]. Mouse models have demonstrated an increase in 12-LOX in diabetes as well as retinopathy of prematurity, which is another retinal neovascular disease [184]. Furthermore, a decrease in VEGF and neovascularization has been shown with an inhibition of the 12-LOX pathway or its deletion [184]. Similarly, 15-HETE has also been shown to increase under hypoxic conditions, upregulate the expression of VEGF, and have pro-angiogenic effects that are inhibited by anti-VEGF antibodies [184,190]. As mentioned previously, PIGF is implicated in pro-angiogenesis and may even trigger the 15-HETE pathway [184]. Lastly, 12-HETE is reported to be increased in the vitreous and retina of humans with PDR, which increases VEGF and induces neovascularization of the retina [13,184,191]. Steroid inhibition of phospholipase A2 and the eicosanoids pathway may prove to have more important roles in PDR management [13].

## 4. Conclusions

The study of the vitreous in animals and humans in vivo, ex vivo, and in vitro has given us great insight into this activity-laden fluidic environment of biology. From the mid-1990s to the present, more advanced vitreous profiling has continued to provide us with more information about the composition of the diabetic vitreous. The generation of newer biotechnologies for vitreous analysis from single-protein studies to multiplex bead analysis has catapulted the efficiency and volume of study in this discipline.

In this review, we discussed the methods of vitreous sampling and techniques and the major proteins and lipid mediators by family in the diabetic vitreous. Although there is still a paucity of information about the communication between critical and minor mediators in the vitreous, there are verifiable conclusions that can be established when viewing the literature as a whole. Growth factors’ correlations, such as VEGF’s, PIGF’s, PDGF’s, FGF-2’s, TGFβ’s, and PEDF’s correlations to DR and to other synergistic vs. antagonistic mediators, have now been greatly elucidated. The major roles of IL-6, IL-8, MCP-1, and ICAM-1 in pro-inflammatory pathways independent of VEGF have now been firmly established as ubiquitous in DR across severity levels. We now also know that the immune system, as well as lipid-based mediators, contributes to disease pathogenesis.

The study of vitreous biology in diabetes allows us to understand the ever-important soup of interaction between innate immunity, protective homeostasis, and destructive inter-connected cascades in diabetic retinopathy. Armed with this growing information, our quest for the identification of preventative and future therapeutic targets is bolstered.

## Figures and Tables

**Table 1:** Selected investigations of major vitreous cytokines in diabetic retinopathy. The location of vitreous sampling is listed if mentioned in the study. The specific diagnoses of the controls are listed if explicitly stated in the study, and if not, they are noted as controls *.

Authors (Year)	Sample Type (s)	Technique (s)	Study Design and Groups	Major Findings
Kauffmann et al. (1994) [121]	vitreous (protein)	ELISA	case series study: 18 PDR (+ VH) no stated controls (detection study)	IL-6 detected (5/18), IL-8 detected (7/18) in PDRv
Elner et al. (1995) [122]	vitreous	ELISA	case–control study: 30 PDR 26 controls (10 cadaveric, 16 including RD, MH, ERM)	↑ IL-8, MCP-1, M-CSF in PDRv
Abu el–Asrar et al. (1997) [123]	vitreous, serum	ELISA	case series study: 42 PDR No stated controls (detection study)	IL-6, MCP-1 detected in PDRv MCP-1 not detected in any serum +IL-6 and MCP-1 vitreous correlation
Yoshida et al. (1998) [64]	vitreous, serum	ELISA	case–control study: 50 PDR (23 quiescent, 27 active) 24 controls *	↑ IL-8 in PDRv (not PDRs); ↑ IL-8 in aPDRv vs. qPDRv IL-8 induced tubule morphogenesis in bovine EC
Kojima et al. (2001) [124]	vitreous, serum	ELISA	case–control study: 30 PDR 10 controls (ERM)	↑ IL-6 in PDRv IL-6 not detected in any serum
Yuuki et al. (2001) [125]	vitreous, serum	ELISA	case–control study: 47 PDR 21 controls (MH)	↑ IL-6, IL-8 in PDRv (not PDRs) ↑ TNFα in PDRs
Nakamura et al. (2003) [126]	vitreous (core cut), serum	ELISA chemiluminescence	case–control study: 62 PDR (34 VH +TRD; 11 VH-TRD; 14 TRD; 3 without VH or TRD) 50 controls (23 MH, 19 RRD, 8 ERM)	↑ pentosidine in PDRv vs. non-diabetic controls ↑ IL-6 in PDRv (>VH groups) No IL-6 elevation in PDRs + correlation pentosidine and IL-6 in PDRv
Funatsu et al. (2003) [83]	vitreous, plasma (protein)	ELISA	case–control study: 26 DME (20 diffuse, 6 cystoid) 12 controls (9 MH, 3 ERM)	↑ IL-6, VEGF in DME (no difference between types) +correlation to each other and hyperfluorescence
Hernandez et al. (2005) [120]	vitreous, serum	ELISA, spectrophotometry	case–control study: 22 PDR (excludes VH) 16 controls (8 ERM, 4 MH, 4 RRD)	↑ IL-8, MCP-1 in PDRv (not PDRs); ↑ aPDRv vs. qPDRv ↓ IL-10 in PDRs vs. controls
Mocan et al. (2006) [27]	vitreous (core), serum	ELISA	case–control study: 8 PDR (VH included) 8 controls (4 MH, 1 IOFB, 1 RD, 2 non-diabetic VH)	↑ IL-6 in PDRv (not PDRs) and in DME
Demircan et al. (2006) [127]	vitreous, serum	ELISA	case–control study: 21 PDR 21 cadaveric vitreous controls 21 serum controls	↑ IL-β, TNFα in PDRv and PDRs vs. cadaveric control vitreous and control serum
Petrovič et al. (2007) [128]	vitreous (mid)	cytometric bead array	case–control study: 71 PDR (48 VH, 17 macular TRD, 6 DME) (excluded VH < 2 months) 17 controls (MH)	↑ IL-8 in PDRv (+ correlation with large vessel obliteration)
Murugeswari et al. (2008) [41]	vitreous, serum	ELISA	case–control study: 25 PDR (25 VH; 16 aPDR; 9 qPDR) 10 controls (MH)	↑ IL-6, IL-8, MCP-1, VEGF in PDRv vs. PDRs IL-1β no difference between PDRv and controls ↓ PEDF in PDRv, PEDF not detected in any serum
Maier et al. (2008) [19]	vitreous, serum	cytometric/multiplex bead array	case–control study: 36 diabetics (16 PDR, 10 NPDR, 10 no DR), VH excluded 69 non-diabetic controls *	↑ MCP-1, IP-10, VEGF in diabetic vitreous + vitreous VEGF correlation with IP-10, MCP-1
Patel et al. (2008) [129]	vitreous	ELISA sandwich enzyme immunoassay	case–control study: 5 PDR (inactive TRD) 15 NPDR 5 controls (MH)	IL-1β detected in PDRv (not in NPDR, controls) ↑ET-1 in PDRv, ↓ in NPDRv ↓ IL-1RA levels (approached significance) in PDRv
Adamiec-Mroczek et al. (2008) [33]	vitreous (needle central), serum	ELISA	case–control study: 46 PDR (43 VH, 3 TRD) 15 controls (8 MH, 4 RD, 2 ERM, 1 dislocated lens)	↑ ICAM-1, VCAM-1, IL-6, TNFα in PDRv and PDRs vs. controls +vitreous correlation between VCAM-1 and TNFα
Yoshimura et al. (2009) [35]	vitreous, serum	ELISA	case–control study: 66 PDR (VH samples excluded) 53 controls (MH, ERM)	↑ IL-6, IL-8, MCP-1, VEGF in PDRv, (+vitreous correlation for all)
Wakabayashi et al. (2010) [130]	vitreous, serum	cytometric/multiplex bead assay	case–control study: 46 PDR (33 active, 13 inactive) 19 non-diabetic controls (13 MH, 6 ERM)	↑ IL-8, MCP-1, IP-10, VEGF, in PDRv (not PDRs) all > in aPDRv vs. qPDRv (except IP-10)
Adamiec-Mroczek et al. (2010) [39]	vitreous (needle central), serum	ELISA	case–control study: 19 PDR 15 controls (8 MH, 4 RD, 2 ERM, 1 dislocated lens)	↑ IL-6, TNFα, ET-1 in PDRv ↑ IL-6, TNFα in PDRs vs. controls +correlation vitreous ET-1 and HgA1C
Suzuki et al. (2011) [131]	vitreous	multiplex bead analysis	case–control study: 63 PDR (35 VH) 23 controls (11 ERM, 12 MH)	↑ IL-6, IL-8, IL-10, MCP-1, PDGF, VEGF in PDRv +correlation of MCP-1, IL-10, IP-10, PDGF to VEGF
Schoenberger et al. (2012) [34]	vitreous	multiplex bead assay, prostaglandin monoclonal EIA kit	case–control study: 26 PDR (VH + TRD) 13 controls (9 MH, 2 ERM, 1 lens dislocation, 1 vitreous opacity)	↑ IL-6, IL-8, MCP-1, TNFα, IP-10, VEGF, PDGF in PDRv ↑ PGE_2_ in PDRv (+correlation VEGF)
Zhou et al. (2012) [40]	vitreous (needle central), serum	ELISA	case–control study: 62 PDR (TRD) 20 non-diabetic controls (12 RD, 8 MH)	↑ IL-1β, IL-6, IL-8, CCL2, endothelin 1, TNFα, VEGF in PDRv IL-10 without change in PDRv
Koskela et al. (2013) [132]	vitreous, plasma	ELISA	case–control study: 39 PDR (11 TRDS) 16 controls (MH + ERM)	↑ IL-6, IL-8, Il-10, sPECAM, sICAM, sVCAM in PDRv vs. controls ↑ IL-6, IL-8 in PDRv vs. PDRp
Gustavsson et al. (2013) [38]	vitreous, serum (protein)	automated chemiluminescence assay, photometry	case–control study: 26 PDR (22 VH, ERM 3, lens material 1) 27 controls (1 VH, 2 ERM, 7 MH, 16 RD, 1 lens material)	↑ IL-6 in PDRv; ↑ IL-6 v/s ratio in PDR ↓ TNFα in PDRv vs. controls ↑ TNFα in PDRs vs. controls
Murugeswari et al. (2014) [24]	vitreous	multiple cytokine biochip, chemiluminescence	case–control study: 13 PDR (VH samples excluded) 5 controls (MH)	↑ IL-6, IL-8, MCP-1, VEGF in PDRv higher IL-6, MCP-1 in vitreous induced endothelial tube formation
Takeuchi et al. (2015) [133]	vitreous (mid) serum	multiplex immunoassay	case–control study: 25 PDR (VH + TRD) 53 controls (27 ERM, 26 MH)	↑ IL-6, IL-17A, TNFα in PDRv vs. PDRs + correlation IL-17A and TNFα
Yoshida et al. (2015) [134]	vitreous	ELISA	interventional case series: 36 PDR before and after vitrectomy (comparison to same eye)	↑ IL-6, IL-8, MCP-1 before vitrectomy in PDRv ↑ IL-6, MCP-1; ↓ IL-8 after vitrectomy in PDRv level of MCP-1 correlated with DME after vitrectomy
Zhao et al. (2015) [135]	vitreous	ELISA	case–control study: 8 PDR (VH, mild stage of PDR) 6 controls (ERM)	Pro-IL -1β, IL-1β not markedly elevated in PDRv vs. controls
Chernykh et al. (2015) [113]	vitreous	ELISA	case–control study: 38 PDR (TRD) 25 controls (non-diabetic TRD)	↑ IL-6, IL-8, IL-17A, PEDF, VEGF in PDRv + correlation of IL-17A, IL-8 to VEGF in PDRv
Chen et al. (2016) [136]	vitreous, blood	ELISA, flow cytometry	case–control study: 30 PDR 30 NPDR 32 controls (MH + ERM)	↑ IL-17A in PDRv and PDRb ↓ IL-17A in PDR PBMCs vs. NPDR PBMCs ↓ Th17 cells in PDR in PBMCs vs. NPDR PBMCs
Yan et al. (2018) [25]	vitreous, serum	ELISA	case–control study: 21 PDR (VH excluded) 32 NPDR 29 controls (ERM)	↓ IL-35 in PDRv vs. controls ↓ IL-27, IL-35 in PDRs vs. controls
Wang et al. (2019) [137]	vitreous, serum, blood	ELISA RT-PCR	case–control study: 20 PDR 20 NPDR 20 controls (8 ERM, 12 MH)	↑ IL-26 in PDRv and PDRs vs. controls ↑ IL-26 mRNA in PBMCs
Shahulhameed et al. (2020) [138]	vitreous, serum (protein)	ELISA Western blotting, gelatin zymography, immunohistochemistry	case–control study: For vitreous: 100 PDR 100 non-diabetic controls *	↑ IL-8, sPECAM, VEGF, MMP9, CHF in PDRv ↓ IL-10 in PDRv ↑ C3bα’ in PDRv ↓ CHF in PDRs vs. NPDR and controls
Suzuki et al. (2020) [30]	vitreous	multiplex immunoassay	interventional case series: 130 PDR (group comparison, no controls) 67 PRP laser prior to vitrectomy 63 PRP intraoperative laser	↑ IL-6, IL-7 intraoperative laser group no difference in VEGF between groups
Urbančič et al. (2020) [43]	vitreous, serum, FVM	cytometric bead array	case–control study: 33 PDR (TRD) 20 controls (MH)	↑ MCP-1, IL-8, VEGF in aPDRv vs. qPDRv and PDRs ↑ IL-6, MCP-1, TNFα, IL-β in PDRv vs. PDRs ↑ T-lymphocytes in FVM

(protein) = vitreous protein concentration was analyzed; VH = vitreous hemorrhage; FVM = fibrovascular membrane; EC = endothelial cell; IOFB = intraocular foreign body; IP-10 = interferon inducible protein 10; PGE_2_ = prostaglandin E2; CCL2 = chemokine (C-C motif) ligand 2; sPECAM = soluble platelet-endothelial cell adhesion molecule; sICAM = soluble intercellular adhesion molecule; sVCAM = soluble vascular cell adhesion molecule; ELISA = enzyme linked immunoassay; EIA = enzyme immunoassay; RT-PCR = real-time polymerase chain reaction; RRD = rhegmatogenous retinal detachment; RD = retinal detachment; MH = macular hole; ERM = epiretinal membrane; PDR = proliferative diabetic retinopathy; PDRv = PDR vitreous; aPDRv = active PDR vitreous; qPDRv = quiescent PDR vitreous; PDRs = PDR serum; PDRp = PDR plasma; PDRb = PDR blood; PBMC = peripheral blood mononuclear cells; M-CSF = macrophage colony stimulating factor; CFH = complement factor H.

## Data Availability

Not applicable.
